# Denervation in Femoral Artery-Ligated Hindlimbs Diminishes Ischemic Recovery Primarily via Impaired Arteriogenesis

**DOI:** 10.1371/journal.pone.0154941

**Published:** 2016-05-13

**Authors:** Yinghuan Cen, Junfeng Liu, Yuansen Qin, Ruiming Liu, Huijin Wang, Yu Zhou, Shenming Wang, Zuojun Hu

**Affiliations:** 1 Department of Vascular Surgery, The First Affiliated Hospital of Sun Yat-sen University, Guangzhou, China; 2 Department of Pathology, The First Affiliated Hospital of Sun Yat-sen University, Guangzhou, China; 3 Laboratory of Department of Surgery, The First Affiliated Hospital of Sun Yat-sen University, Guangzhou, China; University of Illinois at Chicago, UNITED STATES

## Abstract

**Aims:**

Multiple factors regulate arteriogenesis. Peripheral nerves play a crucial role in vascular remodeling, but the function of peripheral nerves during arteriogenesis is obscure. Our study investigated the contribution of denervation to arteriogenesis during post-ischemic recovery from hindlimb femoral artery ligation.

**Methods and Results:**

Sprague-Dawley rats were randomly allocated into four groups of normal control (NC), hindlimb ischemia (HI), hindlimb ischemia with denervation (HID) and hindlimb simple denervation (HD). Hindlimb ischemic recovery was assessed by clinical assessment and tibialis anterior muscle remodeling on day 28 post-surgery. Blood flow was determined by laser Doppler imaging on day 0, 3, 7, 14 and 28 post-surgery. Collateral number of hindlimb was observed by angiography and gracilis muscles were tested by immunostaining on day 7 and 28 post-surgery. Angiogenesis was accessed by counting CD31 positive capillaries in tibialis anterior muscles on day 28 post-surgery. Group HID showed impaired ischemic recovery compared with the other 3 groups and impaired blood flow recovery compared with group HI on day 28 post-surgery. The collateral number and capillary density of group HID were lower than group HI. The collateral diameter of both group HID and group HI significantly increased compared with group NC. However, the lumen diameter was much narrower and the vessel wall was much thicker in group HID than group HI. We also demonstrated that the thickened neointima of collaterals in group HID comprised of smooth muscle cells and endothelial cells.

**Conclusions:**

Denervation of the ligated femoral artery in the hindlimb impairs ischemic recovery via impaired perfusion. The possible mechanisms of impaired perfusion are lower collateral number, lower capillary density and most likely narrower lumen, which damage ischemic recovery. This study illustrates the crucial role of peripheral nerves in arteriogenesis using a model combined ischemia with denervation in hindlimb.

## Introduction

Arteriogenesis or collateral artery growth is a distinct form of angiogenesis, which involves the hypoxia-stimulated outgrowth of capillaries. Arteriogenesis describes the process of pre-existing arterioles maturing into functional arteries via a complex and coordinated process of endothelium activation, monocyte invasion, growth factor and cytokine secretion, matrix digestion and ultimately the proliferation of endothelial cells (ECs) and smooth muscle cells (SMCs), which is triggered primarily by fluid shear stress (FSS) from chronic stenosis or occlusion [1–3]. Many researches have been performed on arteriogenesis, but the underlying mechanisms, especially the role of neural factors, are not fully understood.

Mounting evidence in past decades has demonstrated the close relationship between nerves and blood vessels. It has been found that nerves provide a branching template for arteries to align with, and arterial markers are not expressed unless blood vessels associate with nerves [4]. Nerves and arteries track alongside each other, and they share a set of common signals, such as semaphorins and their receptors, netrins and their receptors, slits and roundabout receptors, and ephrins and Eph receptors, which act as axon guidance cues [5–8]. Other peripheral derived signals, such as chemokine (C-X-C motif) ligand 12 (CXCL12) and vascular endothelial growth factor (VEGF), are also essential for arterial development [9,10]. Functional recovery after ischemia is impaired in apolipoprotein E knockout mice, which exhibits peripheral nerve defects, and this impairment is attributed to the inability to up-regulate VEGF [11]. Several recent studies have focused on the effect of peripheral nerves on angiogenesis in ischemic hindlimbs. Desert hedgehog, which is expressed in the Schwann cells of peripheral nerves, regulates angiogenesis by promoting peripheral nerve survival in ischemic environments in mice [12,13]. Transection of the sciatic nerve with or without hindlimb ischemic surgery reduces capillary numbers, which supports a role for the peripheral nerves in the maintenance and promotion of angiogenesis [14,15]. Ming-ying Luo et al. reported that collateral vessel growth is impaired in a denervated plus femoral artery ligation model, in which the sciatic, femoral, and obturator nerves were resected in succession. The mechanism of this impairment involves reduced adventitia inflammation during an early stage of arteriogenesis [16]. However, the data are scarce and incomplete. We investigated the neural effect on ischemic injury to provide novel data and increase the dataset of vascular studies.

In our study, we generated a model of ischemia and denervation by simultaneously transecting the sciatic, femoral and genitocrural nerves in the presence of femoral artery ligation (FAL) and then examined the hypothesis that denervation would hamper ischemic recovery. We provided evidence that denervation worsens ischemic injury and impairs perfusion recovery, which results from lower collateral number, much narrower lumen and lower capillary density.

## Methods

### Animal model

This study was carried out in strict accordance with the recommendations in the Guide for the Care and Use of Laboratory Animals of the National Institutes of Health. The protocol was approved by the Committee on the Ethics of Animal Experiments of the First Affiliated Hospital of Sun Yat-sen University (Permit Number: [2014] 2). Sixty-six female Sprague-Dawley (SD) rats weighing 200–220 g were randomly assigned to four groups of normal control (NC), hindlimb ischemia (HI), hindlimb ischemia with denervation (HID) and simple denervation (HD). All experimental rats were anesthetized with an intraperitoneal injection of 2% pentobarbital sodium (0.3 ml/100 g). All efforts were made to minimize suffering. Post-surgery, rats were injected subcutaneously with buprenorphine (0.05 mg/kg) to minimize pain and Ampicillin powder (AMRESCO) was applied moderately for external use to the wound twice daily for 3 days. The condition of the rats was monitored twice daily for the first 3 days and once daily for the next 25 days post-surgery by observing the survival rate, appearance and motor function of hindlimbs and intake of food and water. The survival rate of group NC, group HI, group HID and group HD was 9/9, 18/18, 18/20, 18/19 respectively. Rats of group NC and group HI functioned properly and did not showed necrosis of hindlimbs. The remaining rats of group HID and group HD showed severe motor dysfunction and varying degrees of necrosis of hindlimbs. All 63 rats consumed food and water freely.

- **Group HI:** The femoral artery of the right hindlimb of each rat in this group was exposed aseptically through a 1 cm skin incision and gently separated from the femoral vein and nerve. Two ligations were made around the femoral artery: one proximal to the bifurcation of the popliteal artery and saphenous artery and the other distal to the lateral caudal femoral artery (LCFA). The left hindlimb of each rat was subjected to sham operation.
- **Group HID:** Bilateral FAL was performed for this group using procedures identical to group HI. And then the femoral, genitocrural and sciatic nerves on the right hindlimb of each rat were separated, and a 1–1.5-cm piece of nerve was removed. The left hinlimb of each rat was subjected to sham operation of separating above nerves besides FAL.
- **Group HD:** SD rats were prepared, and the right hindlimb of each rat was denervated by removing a 1–1.5-cm piece of the femoral, genitocrural and sciatic nerves following the above-mentioned procedures. The left hindlimb of each leg was subjected to sham operation.

### Clinical assessment

A blinded observer performed clinical assessments 28 days post-surgery to quantify the ischemic injury after femoral ligation and/or denervation. The results were expressed as an appearance score. The following evaluation standards were used for the appearance scores [17]: 0, normal; 1–5, color change in the nail bed or loss of nails, in which the score reflects the number of nails affected; 6–10, partial or complete atrophy of the digit(s), in which the score reflects the number of digits affected; and 11, partial atrophy of the forefoot.

### Laser Doppler perfusion imaging (LDPI)

Superficial hindlimb perfusion was assessed noninvasively pre-surgery, day 0, 3, 7, 14 and 28 post-surgery using a scanning laser Doppler imager (PeriCam PSI, Perimed, Sweden). Rats were fixed in supine position. The hindquarters were depilated before scanning, and the degree of anesthesia and ambient temperature were consistent during scanning. Doppler perfusion of the plantar foot was measured within anatomically defined regions of interest (ROIs). The procedures were described in detail previously [18]. An investigator who was blinded to the subgroups drew all ROIs. The average velocity in an ROI was normalized to the area of the ROI.

### Angiography

SD rats were anesthetized and cannulated via the descending abdominal aorta from the level of the renal artery to the level of the bifurcation of common iliac artery on day 7 and 28 post-surgery. Rats were fixed in supine position. A phosphate-buffered solution (PBS, pH 7.4) containing 20 μmol/L sodium nitroprusside and 20 U/ml heparin was perfused slowly through the abdominal aorta for heparinization and maximal vascular dilation. Iopromide Injection (Ultravist, 370 mg/ml, Bayer) was infused at 0.1 ml/s. X-ray images were obtained in succession for 1 minute using MicroPET/CT (Inveon, SIEMENS). All rats subjected to this experiment were euthanized with an overdose of anesthetic. The number of collaterals connecting distal ligation of the femoral artery through the collateral zone to the posterior edge of the thigh was counted by an observer who was blinded to the subgroups using Image J software.

### Tissue harvesting

Rats were euthanized by an overdose of anesthetic on day 7 and 28 post-surgery. The gracilis and tibialis anterior muscles were removed immediately and cut in half. One half was fixed in paraformaldehyde, embedded in paraffin and cut into 6-μm sections. The other half was snap frozen in liquid nitrogen, fixed in O.C.T. and cut into 6-μm sections. Paraffin-embedded sections were stored at room temperature for later use, and frozen sections were stored at -80°C.

### Histochemical staining and image analysis

Histopathological examinations of hematoxylin-eosin (H&E)-stained gracilis and tibialis anterior muscles were performed in paraffin-embedded sections and photographed under a microscope (Olympus). In total, the number of collaterals traversing gracilis muscles collected for statistical analysis were 8 from group NC, 16 from group HI, 15 from group HID, 8 from group HD (n = 5 per group). Images were processed with software Image J. Sectional areas along with the boundaries of collateral endothelium and collateral adventitia were drawn manually by an observer who is blinded to the subgroups, and expressed as lumen area and total vessel area respectively.

### Immunofluorescence staining

Frozen cross-sections were used in these experiments. Specimens were blocked with 5% BSA for 30 minutes and incubated with a primary antibody for 2 hours at 37°C. Hindlimb nerves were identified by using an anti-mouse S-100 antibody (1:100, Abcam). Vessel smooth muscles were identified using an anti-rabbit α-SM-actin (1:100, Abcam). Capillary ECs and collateral ECs were identified using an anti-mouse CD31 antibody (1:100, Abcam). The appropriate Alexa Fluor® 647 and Alexa Fluor® 488 secondary antibodies (1:500, CST) were used. Nuclei were stained using DAPI. All sections were photographed under a microscope (Olympus).

### Capillary density

Capillary ECs were evaluated 28 days post-surgery in sections of tibialis anterior muscle immunofluorescence stained for CD31 as previously stated. The positively stained capillaries were counted in five random microscopic fields (×200) per slide (n = 5 per group) by a blinded observer, and capillary density was expressed as the number of capillaries/mm^2^ [19].

### Statistical analysis

The results were presented as the mean ± standard error of the mean (SEM). Significance (*p*<0.05) was determined using nonparametric Mann-Whitney tests for group comparisons. Student's t-test (two-sided) was used for intergroup comparisons.

## Results

### Rats of group HID exhibit impaired ischemic recovery after FAL

**Fig 1:**
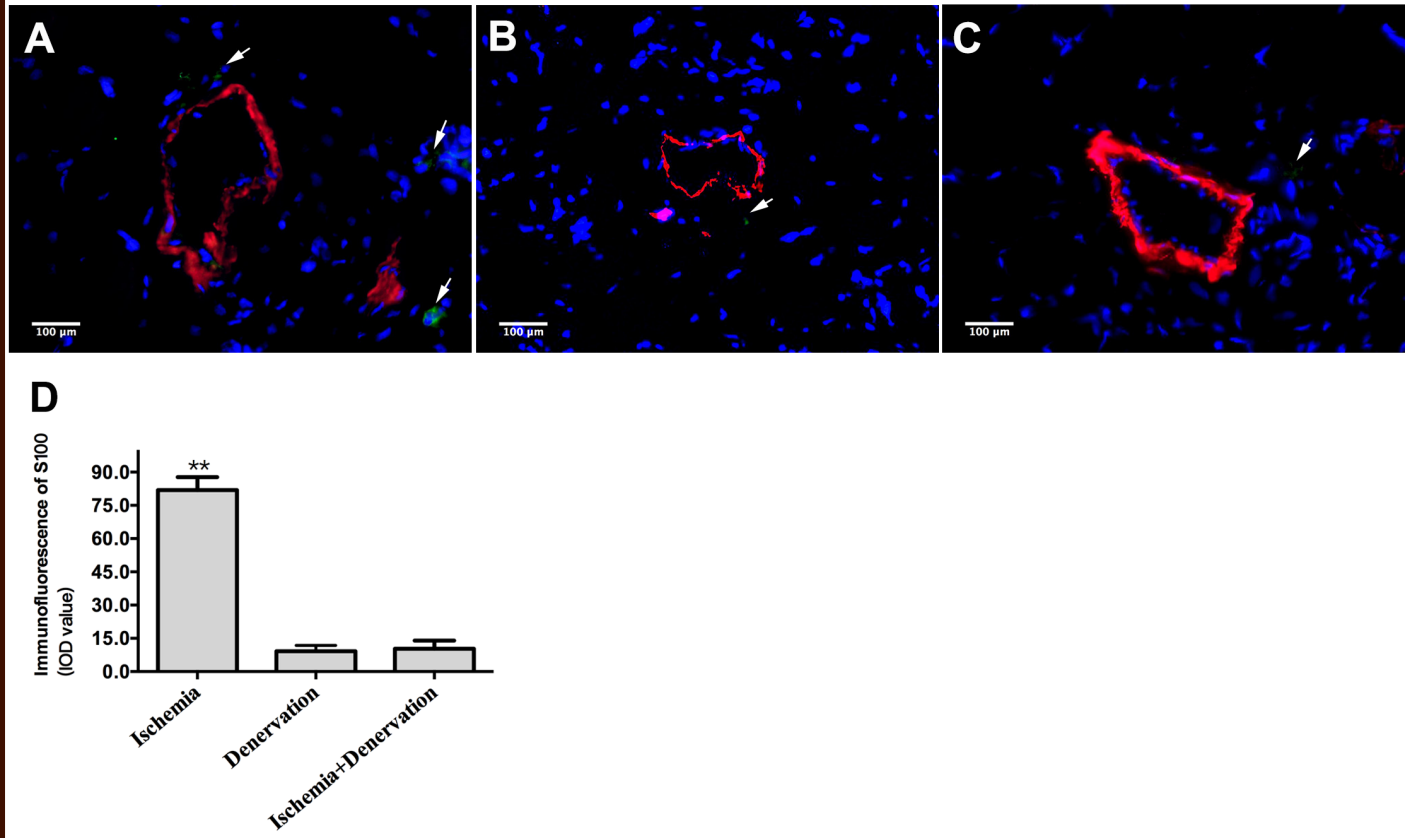
Immunofluorescence staining of the peripheral nerves. The peripheral nerve was identified using S100 and α-SM-actin immunofluorescence dual staining of gracilis muscle cross-sections from group hindlimb ischemia (HI), group simple denervation (HD) and group ischemia with denervation (HID) on day 7 post-surgery. Green indicates S100, red indicates α-SM-actin, and blue indicates nuclei. S100-positive staining which is indicated with white arrows in group HI (A) was significantly higher than both group HD (B) and group HID (C). The S100-positive staining was evaluated as integrity of density (IOD) value (D). ** *p*<0.01.

We removed approximately 1–1.5 cm of the femoral, genitocrural and sciatic nerves immediately after FAL to investigate the role of peripheral nerves in arteriogenesis. We verified that these nerves were almost completely degraded in group HID and group HD compared with group HI on day 7 post-surgery. S100-positive staining was observed in perivascular tissue in group HI (Fig 1A), but little S100 staining was visible in group HD and group HID (Fig 1B and 1C, respectively).

**Fig 2:**
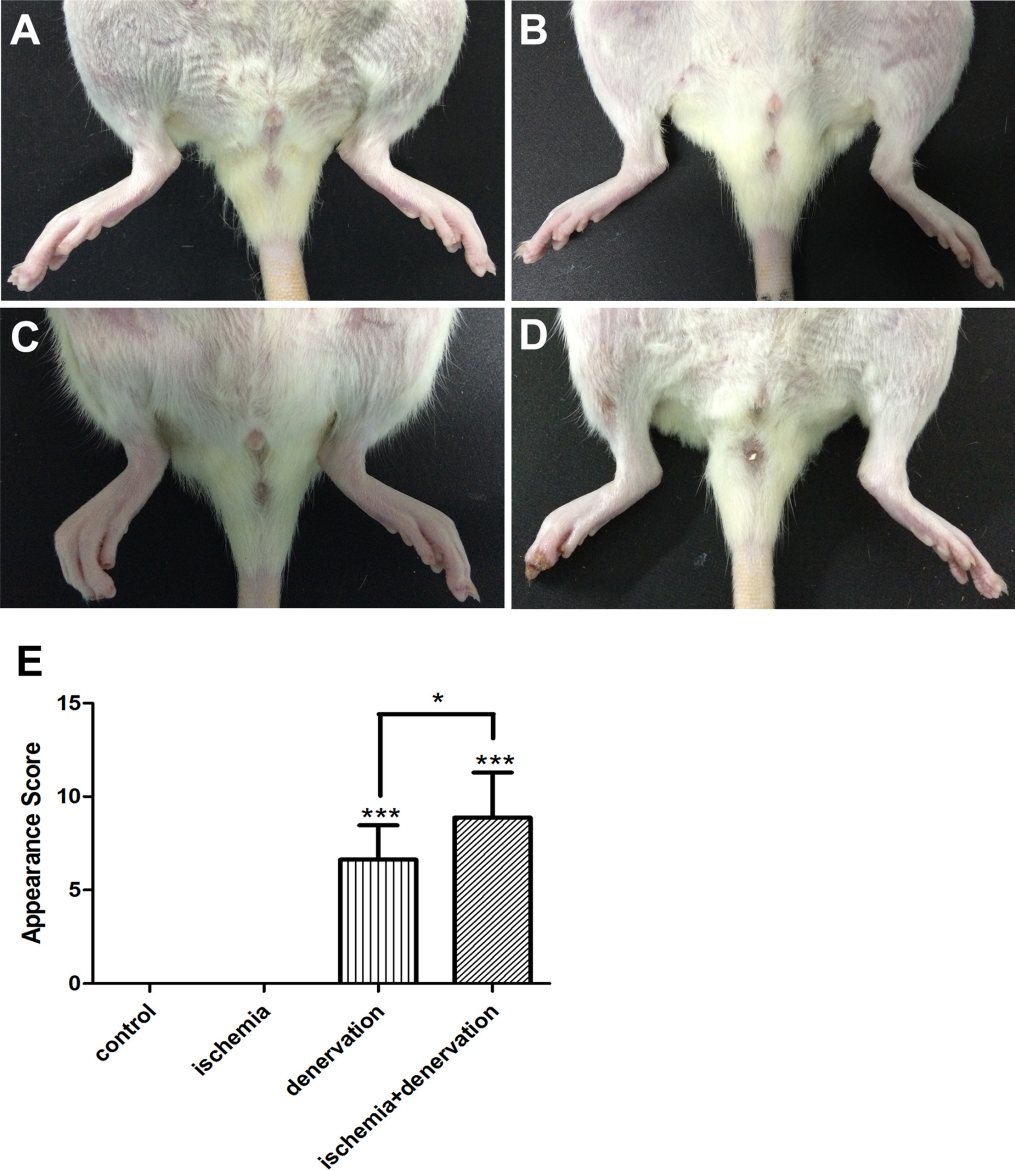
Clinical assessment. Representative images of legs from group NC, HI, HD and HID on day 28 post-surgery. Necrosis was invisible in group NC (A) and group HI (the right leg, B), but it was present in group HD (the right leg, C) and group HID (the right leg, D). Necrosis was even worse in group HID than group HD. The clinical assessment was evaluated as an appearance score (E). * *p*<0.05, *** *p*<0.001.

**Fig 3:**
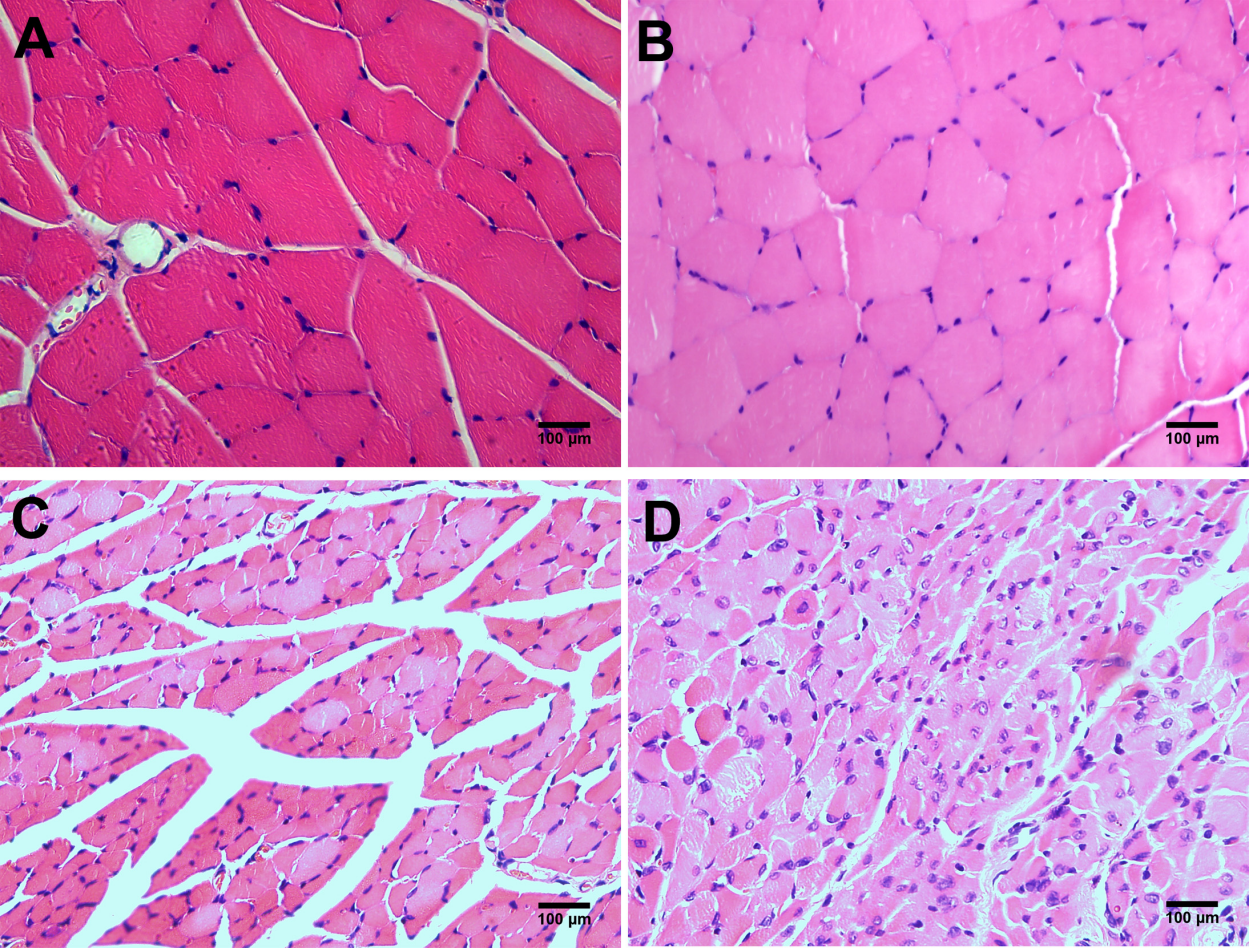
H&E staining of the tibialis anterior muscles 28 days post-surgery. The skeletal muscles were well preserved in morphology and structure, and staining was well distributed in group NC (A) and HI (B). The skeletal muscles were reduced in volume, and the staining was inhomogeneous in group HD (C). The skeletal muscles were reduced in volume severely and disorganized in morphology and structure, and the nuclei were enlarged and located abnormally in group HID (D).

The leg appearance was scored on day 28 using the severity of necrosis, and a distal limb muscle (tibialis anterior muscle) was assessed using H&E staining to evaluate hindlimb ischemic injury. We included group HD as a control of group HID because loss of nerve nutrition would result in skeletal muscle atrophy and necrosis. The mean appearance scores of necrosis were 0 in both group NC and group HI (Fig 2A and 2B), whereas the scores were 6.63±0.65 in group HD (Fig 2C) and 8.88±0.85 in group HID (Fig 2D). Skeletal muscles were well preserved in group NC and group HI (Fig 3A and 3B), which was consistent with the appearance scores. Skeletal muscles underwent degeneration in group HD (Fig 3C) and group HID (Fig 3D). However, muscle fibers exhibited more atrophy and tissue structures were more disturbed in group HID compared with group HD, suggesting that ischemic recovery from ischemic injury is impaired in group HID compared with group HI.

### Rats of group HID exhibit impaired perfusion recovery

**Fig 4:**
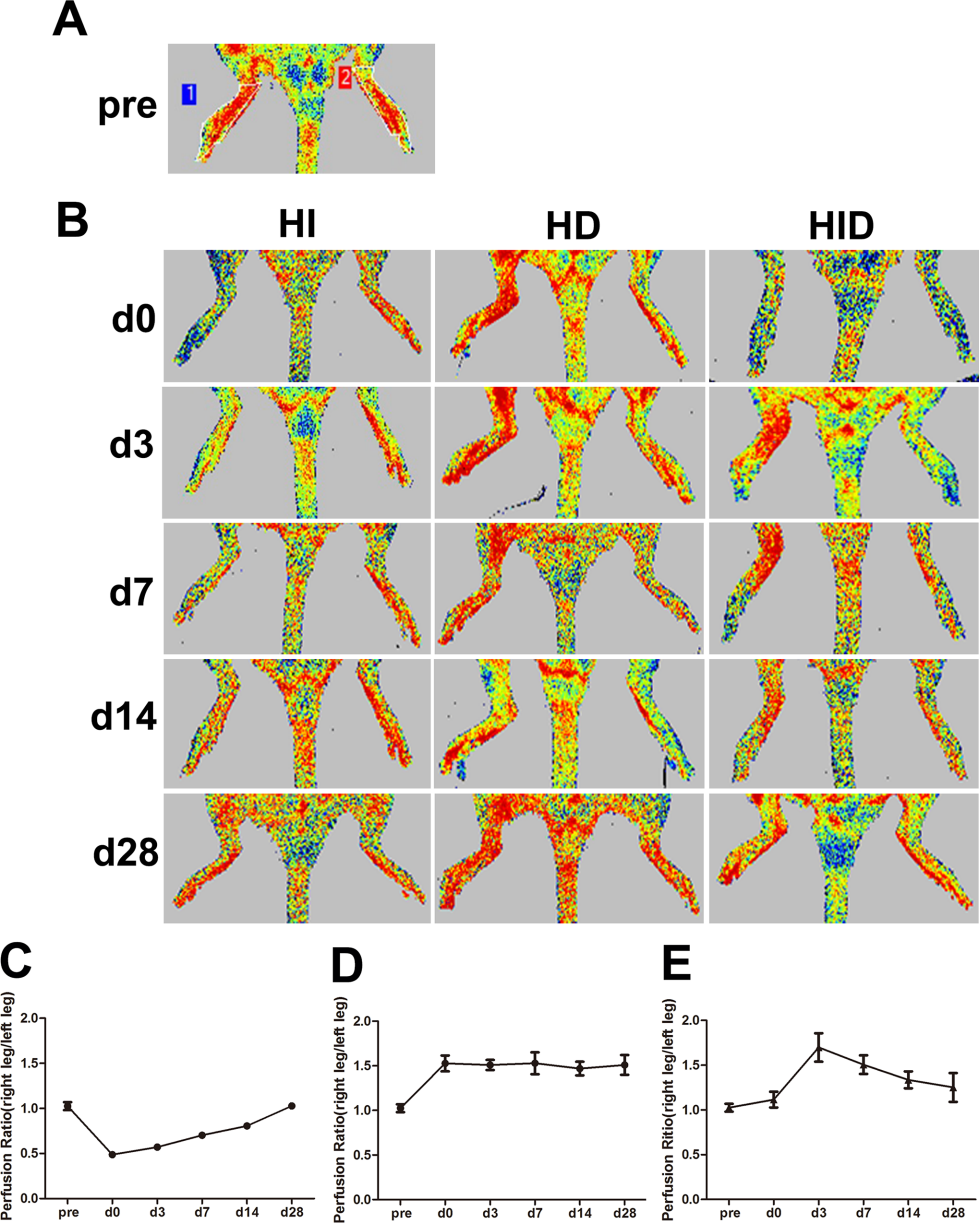
Perfusion recovery. Perfusion recovery is shown by laser Doppler perfusion images (A and B). Perfusion was evaluated in the plantar feet in the same anatomically defined region (shown as A) in all animals (n = 5 per group). The right leg/left leg perfusion ratio of group HI (C), group HD (D) and group HID (E) are shown. In group HI, perfusion ratio fell from 1.02 on baseline to 0.48 immediately post-surgery, but the ratio gradually increased within 28 days and the ratio returned to 1.03 on day 28 post-surgery. In group HD, the perfusion ratio was significantly elevated to 1.47–1.53 immediately post-surgery and each time point within 28 days. In group HID, the perfusion ratio slightly elevated immediately post-surgery and peaked to 1.69 on day 3 but decreased gradually after that and the ratio dropped to 1.25 on day 28 post-surgery.

The hindlimb has several collaterals in the thigh that interconnect the deep femoral artery and LCFA with the popliteal and saphenous arteries. These collaterals undergo remodeling after FAL, and hindlimb perfusion recovers under normal circumstances. Noninvasive laser Doppler imaging (Fig 4A and 4B) was used to measure perfusion in the distal hindlimbs (plantar foot) of the rats. The right limb/left limb perfusion ratio was almost equal at baseline (Fig 4A). Perfusion dropped by ~50% immediately after FAL in group HI, but it increased gradually and recovered well by 28 days (Fig 4C). We also included group HD as a control of group HID to eliminate the effect of vascular tone following nerve sectioning. The right side/left side perfusion ratio of group HD increased by approximately 150% at each time point post-surgey (Fig 4D). The right leg was ischemic and denervated while the left leg was ischemic in group HID. This kind of model provides better comparisons of the effects of denervation and non-denervation on ischemic leg perfusion. The right limb/left limb perfusion ratio of group HID were greater than the group NC at each time point post-surgery, but the ratio peaked on day 3 and dropped after that, and it was ~1.3 on day 28, which was less than the ratio of group HD (Fig 4E). These results suggest that rats of group HID have impaired perfusion recovery.

### Decreased collateral number and capillary density in rats of group HID

**Fig 5:**
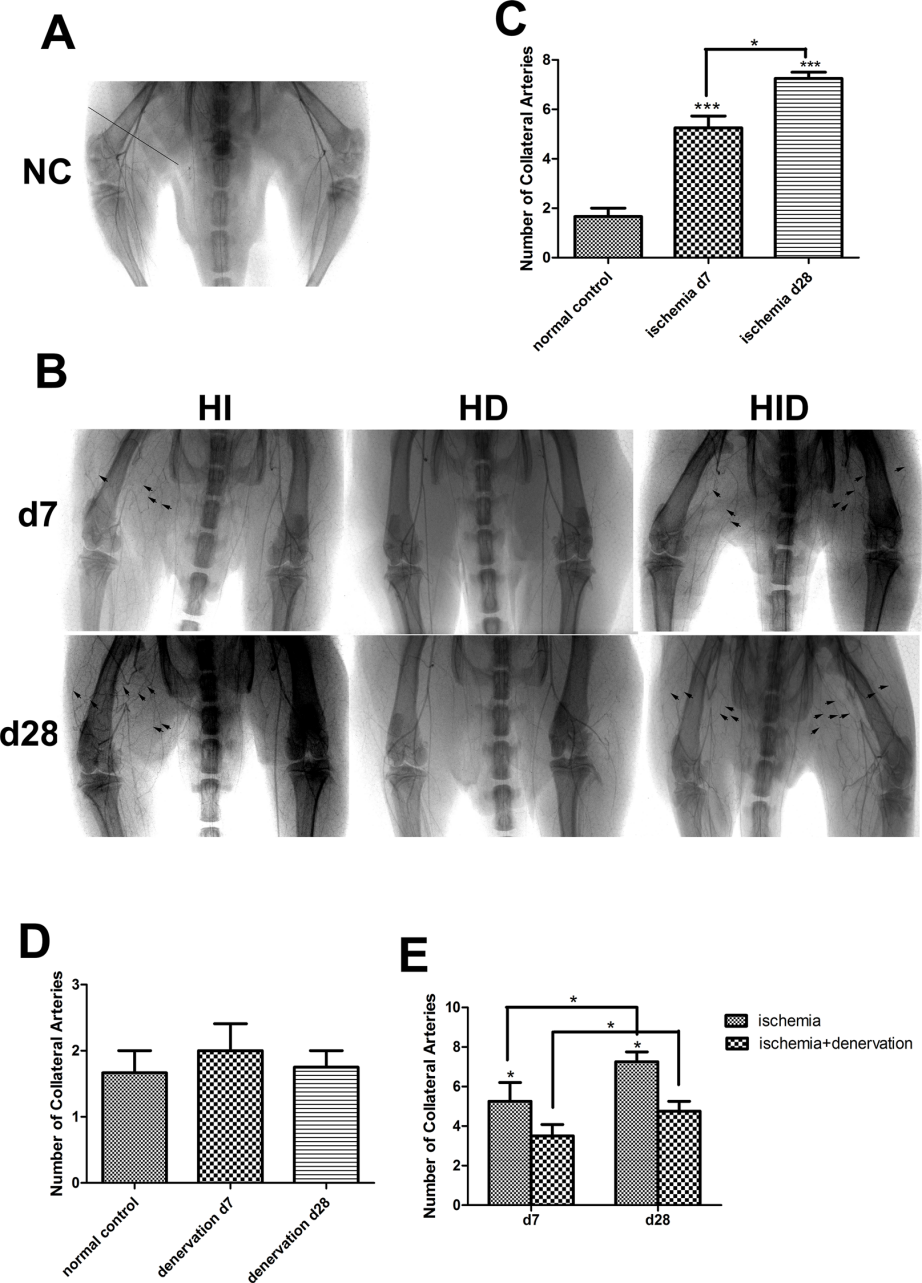
Collateral number shown by X-ray angiograms. The collateral arteries in group NC (A) and HI (right leg), HD (right leg), and HID (right leg: ischemic and denervated, left leg: ischemic) 7 and 28 days post-surgery (B) are shown. The number of collateral arteries was obtained by counting the arteries that intersected a line that was perpendicular to the midpoint of the femur (shown as a straight line in A). The remodeling collateral arteries are indicated with black arrows. Few pre-existing collaterals was seen in group NC (A). The number of collaterals in group HI significantly increased compared with group NC on day 7 and further increased on day 28 post-surgery (C). The number of collaterals did not change 7 or 28 days post-surgery in group HD (D) compared with group NC. In group HID, the number of collaterals (the right leg) also increased compared with group NC, but the number was lower than group HI on day 7 and 28 post-surgery (E). n = 4 per group * *p*<0.05, *** *p*<0.001.

We performed angiography pre-surgery, 7 and 28 days post-surgery to investigate the collateral number (Fig 5). The network of hindlimb collaterals in group NC was not well established. One or two pre-existing arterial collaterals were visible using angiography (Fig 5A), which was consistent with previous studies. The number of visible collaterals increased significantly in group HI on day 7 and further increased modestly on day 28 post-surgery versus group NC (Fig 5C). We measured group HD before performing angiography on group HID to examine whether the collateral number was altered to eliminate possible angiography differences produced by denervation alone. The collateral number in group HD was not different from the group NC on day 7 and 28 post-surgery (*p* = 0.79, Fig 5D). Collateral number was increased in both right (the ischemic and denervated side) and left legs (the ischemic side) of group HID compared with group NC on day 7 and 28 post-surgery. But this increase was less in the right leg compared with the left leg of group HID (3.50±0.29 vs. 5.25±0.33 on day 7 and 4.75±0.25 vs. 7.25±0.25 on day 28, Fig 5E).

**Fig 6:**
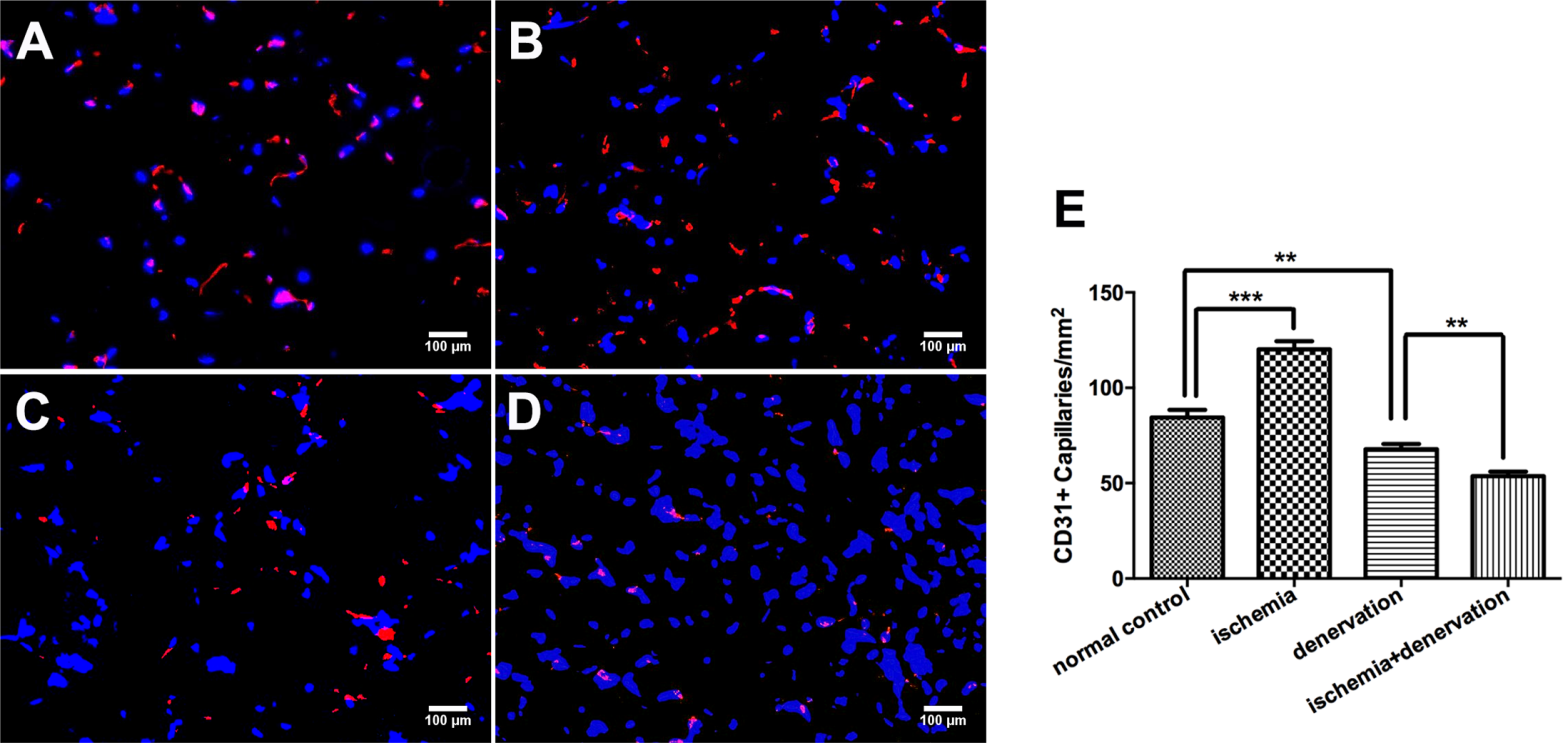
CD31 positive capillary density. Capillary density was evaluated using immunostaining for CD31 of tibialis anterior muscle sections in group NC (A), HI (B), HD (C) and HID (D) on day 28 post-surgery and quantified by counting CD31 positive capillaries per square millimeter. The bar graph shows the capillary density (E). Red indicates CD31, and blue indicates nuclei. ** *p*<0.01, *** *p*< 0.001.

Ischemia-induced angiogenesis was reflected by the capillary density in the tibialis anterior muscle 28 days post-surgery. Capillary density, as visualized with CD31, significantly increased in group HI versus group NC (120.2±4.2 vs. 84.4±4.0 capillaries/mm^2^; Fig 6B and 6A, respectively). Group HD and group HID showed lower capillary density compared with group NC, and the number was even lower in group HID versus group HD (53.7±2.4 vs. 67.8±2.8 capillaries/mm^2^; Fig 6D and 6C, respectively).

### Collaterals from group HID exhibit a narrower lumen and thicker intima

**Fig 7:**
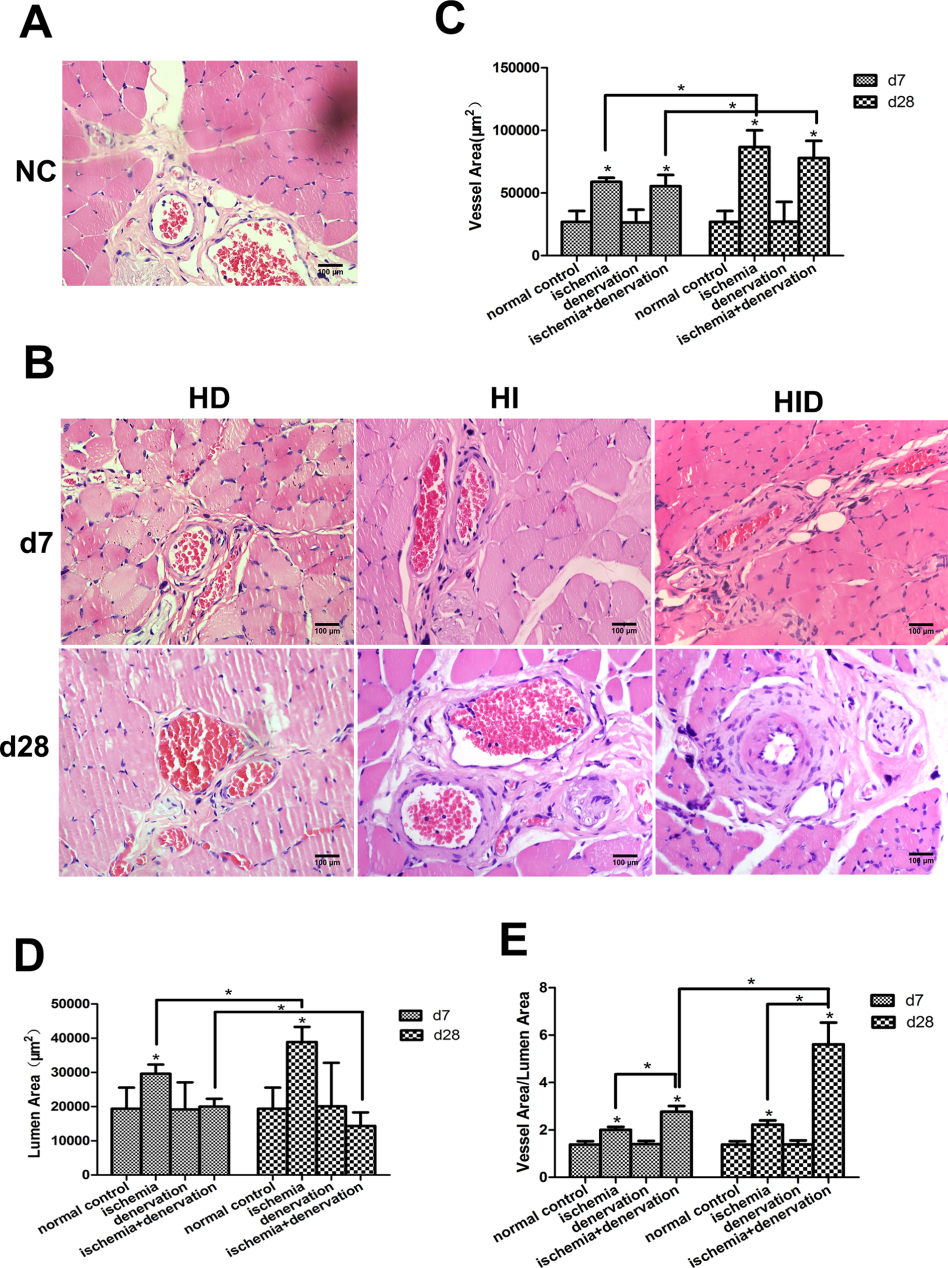
Cross-sections of gracilis anterior muscle were stained with H&E. Representative photographs are shown (A and B). The collaterals were quantified by vessel area (C), lumen area (D), and vessel area/lumen area (E). There were no differences in vessel area, lumen area or vessel area/lumen area of group HD compared with group NC. In group HI and HID, the vessel area increased 7 days post-surgery and further increased 28 days post-surgery. However, in comparison with group NC, the lumen area increased significantly and vessel area/lumen area increased modestly in group HI, but the lumen area decreased and vessel area/lumen area increased dramatically in group HID. * *p*< 0.05.

H&E staining was performed on the gracilis muscles on day 7 and 28 post-surgery to investigate the collateral remodeling process by evaluating vessel area, lumen area and vessel area/lumen area of the collaterals (Fig 7). In group HI, it increased 2.19-fold in vessel area, 1.5-fold in lumen area and 1.45-fold in vessel area/lumen area on day 7 compared with group NC. These changes were further developed on day 28 post-surgery. Collaterals in group HD were not different from group NC (*p*>0.05), which suggests that collateral remodeling requires the onset of FSS created by artery ligation. No significant difference was observed in vessel area in group HID compared with group HI (*p* = 0.19), but the lumen area was much smaller (20000.15±811.48 vs. 29586.07±957.4 μm^2^ on day 7 and 14335.51±1404.62 vs. 38910.34±1555.96 μm^2^ on day 28, respectively). The vessel wall (vessel area/lumen area) of group HID was much thicker compared with group HI (2.79±0.09 vs. 2±0.05 on day 7 and 5.61±0.32 vs. 2.22+0.06 on day 28, respectively), which indicates active cell proliferation in the vessel wall.

### SMC and EC proliferation contribute to wall thickness and lumen narrowing in rats of group HID

**Fig 8:**
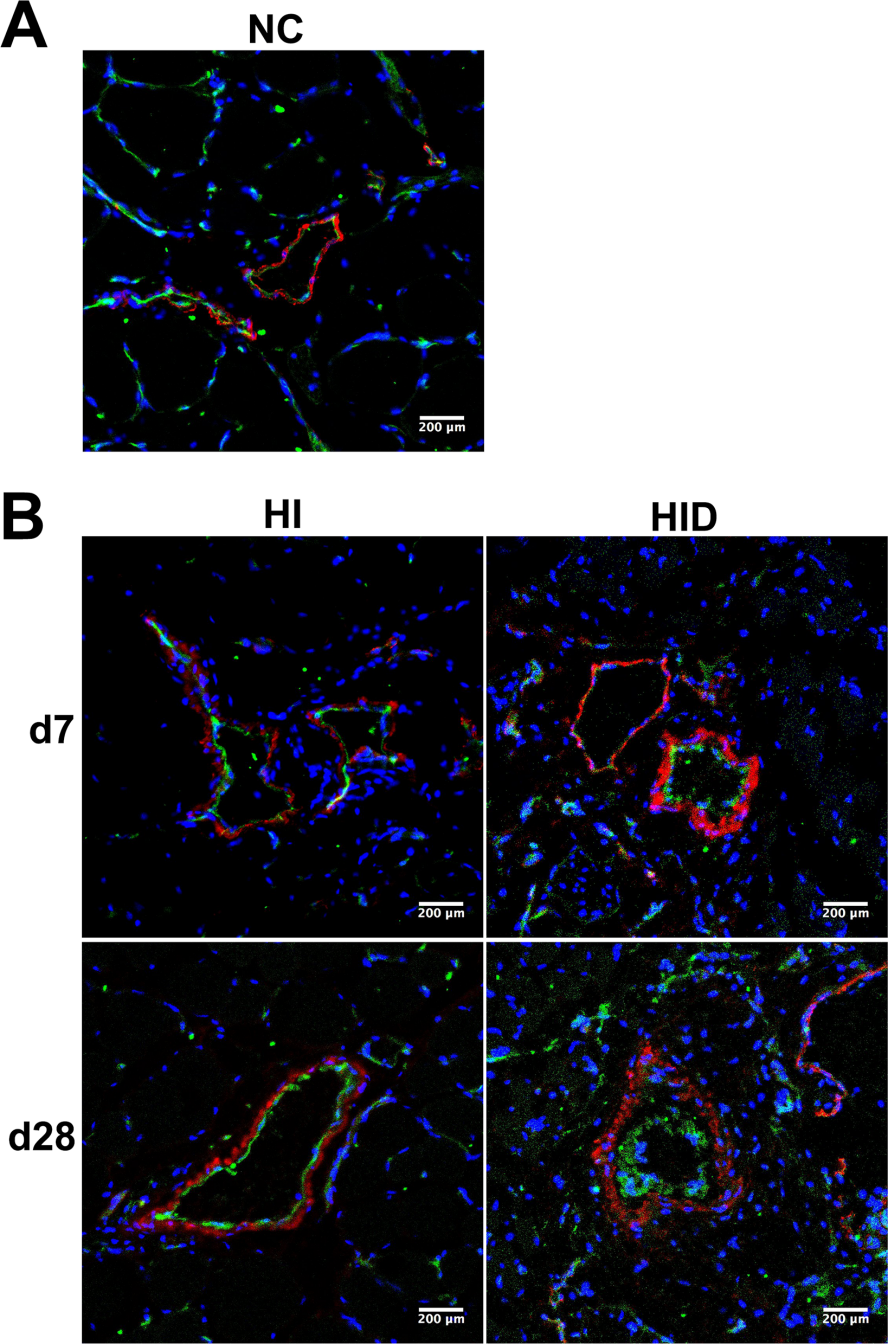
SMCs and ECs staining of collaterals in cross-sections of gracilis muscle. Dual immunostaining of ECs with CD31 and SMCs with α-SM-actin in cross-sections of gracilis muscle in group NC, HI, HID. A thin layer of CD31 staining was observed in the collaterals of group NC (A) and HI on day 7 and 28 post-surgery, but the CD31 layer was much thicker on day 7 post-surgery and further thickened dramatically on day 28 post-surgery in group HID compared with group NC and HI (B). Similarly, the layer of α-SM-actin staining was thin in the collaterals of group NC (A). In group HI, the layer of α-SM-actin staining increased compared with group NC on day 7 and further increased slightly on day 28 post-surgery. In group HID, the staining layer increased significantly on day 7 and much further increased on day 28 post-surgery (B). CD31 indicates ECs (green), α-SM-actin indicates SMCs (red), and DAPI indicates nuclei (blue).

Vessel SMCs and ECs were identified in gracilis cross-sections using immunostaining of α-SM-actin and CD31, respectively, on day 7 and 28 post-surgery (Fig 8). No significant difference in the layer of ECs was observed between group HI and group NC 7 days or 28 days post-surgery. However, the layer of ECs grew thicker in group HID compared with other groups on day 7, and it was much thicker on day 28 post-surgery. Similarly, the layer of SMCs was slightly thicker in group HI compared with group NC. By comparison to other groups, it was much thicker in group HID on day 7, and the thickness further increased on day 28 post-surgery.

## Discussion

Numerous studies use FAL to induce hindlimb ischemia as a model to study the mechanism of arteriogenesis. To the best of our knowledge, the present study is the first to demonstrate the crucial role of peripheral nerves in arteriogenesis by combining FAL and the simultaneous resectioning of the femoral, genitocrural and sciatic nerves in one hindlimb of rat. Ming-ying Luo et al. [16] used a similar model in which the femoral artery was ligated and the femoral and obturator nerves were resected one week after the sciatic nerve was resected. We assumed that resection of the sciatic nerve one week in advance would create a different pathophysiological environment from the control models [20], and the time span of these experiments was not equal. We investigated whether the nerves remained completely resected or regenerated 7 days post-surgery to ensure that the model was successful.

The following changes are primarily identified in group HID: (1) worse ischemic recovery; (2) impaired perfusion recovery; (3) fewer collateral numbers, extremely narrow lumen and intima hyperplasia, and impaired angiogenesis (as the three mechanisms of impaired perfusion recovery); and (4) SMCs and ECs contribute to intima hyperplasia. These findings suggest that peripheral nerves are an indispensable factor for arteriogenesis following FAL in the rat hindlimb.

Surgical denervation damages all nerve fibers, including sensory, motor, sympathetic and parasympathetic nerves [21]. It is widely recognized that sympathetic nerve maintains vascular tone by its release of noradrenaline, adenosine-triphosphate and neuropeptide Y [22–24]. Loss of sympathetic innervation causes dilation of vessels, which are suppliing the muscles and skin. This effect may explain the elevated perfusion observed with laser Doppler imaging as seen in both group HD and group HID. Strangely, the perfusion of the denervated with ischemic legs were not markedly but slightly higher than the ischemic legs immediately post-surgery. The main reason to that phenomenon was probably that it took more time and anesthetic dosage to perform the complicated surgery in group HID resulting in low blood flow of limbs immediately post-surgery. Sympathectomy or sympathetic blockage could usually be used as a method to increase blood flow to improve symptoms such as ischemia [25,26]. Though the similar phenomenon of elevated perfusion is observed in group HID, the ratio to group HI is gradually declining and the red signal is weaker than group HD, suggesting that the effect of the elevated perfusion brought by sympathectomy has been neutralized by impaired arteriogenesis. The elevated perfusion in group HID is slight and is tended to decrease. Actually, due to huge molecular changes and apoptotic reaction, denervation is an energy-consuming process [27]. After denervation, cell death occurs with the denervated muscle fibers undergone atrophy and structural changes[28]. Therefore, the group HD and the group HID exhibited worse necrotic appearance and muscle fiber remodeling compared with non-denervated muscles and these conditions were even worse in group HID. These results indicate that the slight elevated perfusion may be not enough for that consumption of energy, thus aggravating the foot necrosis to some extend.

The group HI never presented necrosis over 28 days post-surgery, and the muscle fibers were integrated, which indicates that the distal muscle rarely suffers from ischemia 28 days post-surgery. Femoral ligation site selection is important. A ligation site distal to the LCFA and proximal to the bifurcation of popliteal and saphenous arteries is the classical ligation site, which recruits invariable collaterals in the gracilis muscle for collateral analyses and causes moderate ischemia in and below the calf [17]. A severe ischemia model is generated using FAL as high as the inguinal canal, and this model may be used to study ischemia of a collateral region, but it is difficult to identify collaterals for histomorphometric analyses [17]. The simultaneous denervation of sciatic, femoral and genitocrural nerves could greatly harm the hindlimb. Therefore, we used a moderate ischemic model and only ligated the femoral artery, without ligation of its branches, to increase limb survival.

We measured capillary density, collateral number and collateral morphological structure which are the three factors that may explain the impaired perfusion in group HID. Angiogenesis is measured by the counting of capillaries per muscle fiber or capillaries per millimeter [19,29]. We used capillaries per millimeter to quantify capillary density because denervation of the hindlimb could cause skeletal muscle degeneration and structural disorganization. Capillary number was decreased 28 days after denervation as a result of their degeneration and atrophy which is similar to previous studies [12,14,15]. However, in group HID, capillary density was lower than group HD. According to previous study, progressive interstitial fibrosis and collagen deposits separate capillaries from each other, which may be one of the reasons of decreased capillaries[15]. As we could see in Fig 3, the interstitial fibrosis resulted from ischemia was more severe in group HID than that in group HD, therefore, we assume the interstitial fibrosis caused lower capillary density in group HID. On the contrary, the capillary density was greatly increased in group HI. Impaired perfusion recovery may be attributed to capillary loss to some extent. However, collateral growth may markedly restore blood flow, and capillaries cannot increase perfusion if the upstream blood flow is limited [30]. So we consume that angiogenesis plays small part in impaired perfusion in our case. Because arteriogenesis outweighs angiogenesis in the recovery of FAL perfusion, we focus on arteriogenesis. The restoration of blood flow of occluded artery lies mostly on collateral number and collateral morphological structure. In normal rats, one or two pre-existing collaterals regularly traverse the collateral zone. After FAL, the collateral number increases significantly, but the angiographically collateral number of group HID is less than group HI. Collaterals normally undergo morphological and structural transformations after FAL and exhibit larger lumens and reasonably thicker walls than normal controls. By contrast, the collaterals within the gracilis muscles in group HID exhibited narrow lumens 7 days post-surgery and extremely narrow lumens 28 days post-surgery, and the vessel wall thickness was higher 7 days post-surgery and extremely higher 28 days post-surgery. These results indicate very active cell proliferation in the vessel wall. Furthermore, the lumen area was even smaller than pre-existing collaterals, with a very thick intima, which resulted in a high resistance for blood. Taken together, these results greatly support that arteriogenesis is impaired by peripheral denervation.

There are no direct proofs for the mechanisms of impaired arteriogenesis induced by denervation. A few hypotheses have been suggested. One of the hypotheses is the alternation of vascular tone of denervated arteries. This hypothesis is founded on the notion that changes in vascular tone produce alternation of FSS acting on endothelium, the initiation of arteriogenesis [31]. FSS is the tangential pressure produced by friction of the blood exerting parallel to the vessel wall [32]. It has long been accepted that vessel remodeling is determined by blood flow and there exists a FSS set point [33]. With increased or decreased flow vessels remodel outwardly or inwardly respectively, to adjust lumen diameters accordingly. However, blood flow is not the only factor determining FSS, among them include vessel resistance. Vessel resistance is the sum of contractile status of arteries (active stiffness) and vascular wall (passive stiffness) [34]. Previous studies have proposed that an increase in sympathetic nerve activity is associated with greater shear stress [35]. On above basis, it is conceivable that denervation changes the vessel tone and causes lower FSS in the FAL hindlimb though the blood flow will increase accordingly as observed in group HID of our study. To examine the correlation between shear pattern and atherosclerosis, Caro CG et al. found that low shear regions show greater burden of atherosclerotic lesions than high shear regions [36]. Similarly, other researchers reported that decreased shear stress is positively correlated with intima thickening in atherosclerosis [37]. Thus, we hypothesize that the decreased FSS in group HID could most likely be an critical contributor to intima hyperplasia, causing collateral stenosis which impaired the blood perfusion. Additional research is needed to investigate the alternation of FSS during arteriogenesis after denervation.

Another hypothesis is that sympathetic innervation functions in stabilizing vascular wall cells phenotype. Some cases of patients with hypertension treated with renal sympathetic denervation have been reported to present neointimal stenosis of renal arteries [38–40]. Neointimal thickening is observed after sympathetic denervation either in humans or in animal models. Rabbit models undergone sympathectomy manifest thickened intima formation, with increased dedifferentiation of SMCs in the media and increased vimentin expression, a maker of immature phenotype of SMCs [41]. It has been showed by others researchers that in sympathectomized vessels both SMCs and fibroblasts increase in numbers, and relate to collagen III up-regulation and simultaneous collagen IV down-regulation which is a phenomenon seen in atherosclerosis plaque formation [42]. These observations suggest that sympathetic nervous system protects from phenotypic switch of vascular wall cells towards dedifferentiated phenotype and sympathectomy induces those cells dedifferentiation and active proliferation. Similar to previous studies, denervation plus FAL induces cell hyperplasia in vascular wall, including SMCs and ECs proliferation in our results. Our results also show that there are no intimal thickenings by denervation alone as in group HD, suggesting sympathetic denervation alone is not sufficient to induce morphological changes of collaterals. It is likely be intertwined with other factors like hemodynamic changes induced by FAL.

Inflammatory reaction, an indispensible process of arteriogenesis can induce numerous proangiogenic and proarteriogenic factors in most cases. So we hypothesis there may exist aberrant inflammatory reaction in arteriogenesis by denervation. Activation of the endothelium by FSS induces the expression of multiple genes during arteriogenesis, including genes that attract inflammatory cells, which produce proteases to break down internal elastic laminae and the extracellular matrix and opens the barriers of EC and SMC proliferation and migration [43]. The vessel diameter of collaterals in group HID increased with a narrow lumen, which indicated that protease activity was not weakened and the inflammatory reaction was not impaired. Nerves undergo Wallerian degeneration followed by inflammation after denervation. There is a significant increase in proinflammatory mediators, such as TNF-α, MCP-1, MIP-1α, IL-1α and IL-1β, at the site of degeneration [44,45]. Massive macrophages are activated from resident cells and the circulatory system, and this activation peaks between days 3 and 14 after nerve injury [46]. The vast number of growth factors that are secreted by the accumulating macrophages due to Wallerian degeneration may cause the over-proliferation of SMCs and ECs.

In conclusion, denervation impairs arteriogenesis after FAL in rat. Our study investigated the role of peripheral nerves in arteriogenesis. We did not provide direct evidence that peripheral nerves act positively on collateral artery growth, but we denervated the area and showed impaired collateral remodeling instead. The mechanisms of SMC and EC proliferation are not known, but they may be investigated in our following research during the process of arteriogenesis, which includes endothelium activation, inflammatory reactions, protease activation, basal membrane degradation and growth factor secretion.

## Abbreviations

NC: normal control
HI: hindlimb ischemia
HID: hindlimb ischemia with denervation
HD: hindlimb simple denervation
EC: endothelial cell
SMC: smooth muscle cell
FAL: femoral artery ligation
LCFA: lateral caudal femoral artery
FSS: fluid sheer stress
